# Effectiveness of auricular acupuncture combined with nicotine replacement therapy for smoking cessation

**DOI:** 10.18332/tid/94328

**Published:** 2018-09-10

**Authors:** Sangho Hyun, Hyuk Huh, Nam gyu Kang

**Affiliations:** 1 Republic of Korea Air Force, Chungju, Republic of Korea

**Keywords:** auricular acupuncture, tobacco abstinence, nicotine replacement therapy, complementary therapy

## Abstract

**INTRODUCTION:**

The aim of this study was to establish if the auricular acupuncture treatment can play a complementary role in enhancing the smoking cessation rate of the smokers receiving conventional therapy, such as NRT and behavioral counseling.

**METHODS:**

This was a retrospective cohort study. Republic of Korea Air Force soldiers who visited the clinic with the intention to quit smoking from September 2016 to March 2017 were reviewed. The smoking cessation program consisted of a 6-weeks treatment period with follow-up at 3 and 6 months from the baseline. Smokers who chose to receive nicotine replacement therapy (NRT) were compared with those who chose to receive auricular acupuncture combined with NRT. Both groups received behavioral counseling.

**RESULTS:**

A total of 148 subjects were reviewed in the study. Of the 86 smokers who received combination therapy, 41 achieved continuous abstinence (47.7%), while 19 out of the 62 smokers stayed abstinent from the NRT group (30.6%). There was a significant difference between the two groups (p=0.037). Minnesota Nicotine Withdrawal Scale scores significantly decreased after the treatment in both groups, but there was no significant difference between the two groups (p=0.681). No serious adverse events were reported from both groups.

**CONCLUSIONS:**

The results of this study indicate that conventional treatments, such as NRT and behavioral counseling, when combined with auricular acupuncture could be a safer and more effective smoking cessation treatment than conventional treatments alone.

## INTRODUCTION

Cigarette smoking is the main cause of preventable deaths, responsible for approximately 6 million deaths worldwide annually^1^. Major causes of smoking induced mortality are cardiovascular diseases, lung cancer, and chronic obstructive pulmonary disease^2-4^. Therefore, smoking cessation can lead to substantial health benefits, and quitting smoking before the age of 40 years is associated with a large decline in the development of smoking related diseases^5^. However, in attempting self-cessation without treatment, the rate of relapse was more than 60% within two weeks, and only about 5% were successful in cessation^6^. Nicotine replacement therapy (NRT) was found to be superior to a placebo, increasing abstinence rate up to twofold^7^, and the efficacies of bupropion and varenicline have been also proven through randomized trials^8^. Although some side effects occur, such as nausea, insomnia, and headache, the consensus among experts is that NRT, bupropion and varenicline are the first-line pharmacological therapies for smoking cessation today^9,10^.

There has been an effort to increase the abstinence rate to help people who wish to quit smoking. A novel approach, such as using nicotine vaccine^11^, and combining two or three of the first-line agents, has been tried^12,13^, but each had drawbacks. The new pharmacological agent showed less or uncertain safety and efficacy compared with the first-line agents, and the combination therapy of the first-line medications displayed more side effects. While trying to find a safer way to increase the rate of smoking cessation, it was clear to us that the effectiveness of auricular acupuncture treatment as a complementary therapy has never been studied. Although it failed to show consistent results in the researches verifying its efficacy for smoking cessation, auricular acupuncture proved its safety in many previous studies^14-17^.

The aim of this study was to establish if the auricular acupuncture treatment can play a complementary role to help enhance the smoking cessation rate of the smokers receiving conventional therapy, such as NRT and behavioral counseling. To achieve this, we reviewed the smokers who visited the clinic to quit smoking, and compared the combination therapy group and the NRT group. Both groups received NRT and behavioral counseling. In addition, we evaluated the satisfaction of the smokers who experienced auricular acupuncture treatment. We consider this study as a preliminary study for more detailed future studies verifying the effectiveness of auricular acupuncture as a safe complementary therapy for smoking cessation.

## METHODS

### Study design

This is a retrospective cohort study. Republic of Korea Air Force soldiers who visited the medical clinic of the Aviation Medical Battalion with the intention to quit smoking from September 2016 to March 2017 were reviewed. Our smoking cessation program consists of a 6-weeks treatment period with follow-up at 3 and 6 months from baseline. Smokers chose the type of treatment that would help them quit smoking after counseling. Smokers who chose to receive NRT were compared with those who chose to receive auricular acupuncture combined with NRT.

Smokers who have been smoking more than 10 cigarettes per day for more than 6 months were included, while smokers with a history of cerebrovascular diseases, heart diseases, hypertension, diabetes, substance abuse other than tobacco, and mental illness were excluded. Also, smokers who were currently receiving other treatments for smoking cessation at the baseline were excluded from the study.

The study was conducted according to the principles of the Declaration of Helsinki. The study protocol was approved by the Institutional Review Board of the Armed Forces Medical Command of Republic of Korea (AFMC-18002-IRB-18-003).

### Interventions

During the 6-week treatment period, the smokers visited the clinic once a week. All smokers included in this study received NRT. They were provided with nicotine patches (Nicotinell, Novartis, Switzerland) and nicotine gum (Nicotinell gum, Novartis, Switzerland). One nicotine patch was applied every morning, and the attachment site was altered every day. Those who smoked more than 20 cigarettes per day were given Nicotinell TTS 30 (nicotine absorption rate of 21 mg in 24 h), and those who smoked less than 20 cigarettes per day applied Nicotinell TTS 20 (nicotine absorption rate of 14 mg in 24 h). Nicotine gum contained 2 mg of nicotine, and the smokers were allowed to chew up to 15 pieces per day^10^. The smokers in both groups did not use NRT after the 6-week treatment period.

All subjects received behavioral counseling. Behavioral counseling was provided by a Republic of Korea Air Force nursing officer who received education on smoking cessation counseling. The counseling was based on the 5As counseling process in the United States Heath Care guidelines^18^. The 5As strategy comprises: ‘Asking about the smoking status’, ‘Advising smoking cessation’, ‘Assessing the will to smoking cessation’, ‘Assisting smoking cessation’, and ‘Arranging a follow-up visit’. The subjects received 5 to 10 minutes of counseling once a week during the 6-week treatment period.

The combination therapy group received auricular acupuncture treatment six times, once a week during the 6-week treatment period. The acupoints of ‘Shenmen’, ‘Lung’, ‘Mouth’, ‘Inner nose’ and ‘Endocrine’ were used, as suggested by the guideline on acupuncture treatment for smoking cessation developed by the Association of Korean Medicine^19^. Sterile intradermal acupuncture needles with a diameter of 0.2 mm and a length of 1.5 mm (Dong Bang, South Korea) were applied on the acupoints, alternating right and left sides every week to avoid constant stimulation of the same site that can lead to infection. Intradermal needles are combined with tapes, so that as they are inserted into each site, the tape naturally covers the site. Acupoints were needled after disinfection, and the technique was performed by a qualified Korean medical doctor with more than 6 years of clinical experience. The subjects were instructed to remove the intradermal acupuncture needles by taking off the tapes covering them when the needles stayed on for more than three days. After removing the needles or if the needles fell off during the 3-day period, the subjects were advised to keep the area clean to prevent infection. The smokers were instructed to stimulate the needle-inserted acupoints by pressing on them when there was a desire to smoke.

### Measures

The primary outcome of this study was the abstinence rate. We checked the exhaled carbon monoxide (CO) level every week during the 6-week treatment period, with an exhaled CO level lower than 7 ppm indicating abstinence. Urine cotinine level was examined by using NicoSign (Maxhealth, South Korea) at 3 and 6 months from the baseline, with a level lower than 200 ng/mL indicating abstinence^20^. Smokers who did not show up for the next treatment or the follow-up were considered to have failed to quit smoking.

The secondary outcome was the nicotine withdrawal symptoms evaluated by the Minnesota Nicotine Withdrawal Scale (MNWS)^21^. The MNWS scores were assessed at the baseline, at the end of the treatment. All reported adverse events were collected from both the NRT group and the combination group, and the satisfaction with auricular acupuncture treatment was checked for the combination treatment group.

### Statistical analysis

Statistical analysis was performed with the Statistical Package for the Social Sciences version 18.0 for Windows. We used independent t-test for the continuous variables and chi-squared test for the categorical variables to examine the significant differences between the NRT group and the combination therapy group. Two-sided p-values less than 0.05 were considered to indicate significant differences. Paired t-test was used for the comparison between the MNWS scores before and after the treatment.

## RESULTS

Comparisons of the baseline characteristics between the NRT group and the combination of NRT and auricular acupuncture treatment group are shown in Table 1. There were no significant differences between the two groups at the baseline.

**Table 1:** Baseline characteristics of the treatment groups (N=148)

*Characteristics*	*Nicotine replacement therapy group (n=62 )*	*Combination therapy group (n=86 )*	*p*
Age (years)	21.6 ± 1.1	21.9 ± 1.4	0.209
Sex (% male)	62 (100.0)	86 (100.0)	
Height (cm)	174.6 ± 5.0	174.4 ± 5.1	0.789
Weight (kg)	68.5 ± 8.4	68.4 ± 8.4	0.959
Body mass index (kg/m^2^)	22.4 ± 2.4	22.5 ± 2.5	0.928
**Smoking history**			
Years smoked	4.3 ± 2.5	4.4 ± 2.4	0.800
Cigarettes a day	13.2 ± 4.1	12.9 ± 3.4	0.569
**Questionnaires**			
FTND score	3.7 ± 1.9	3.5 ± 1.8	0.615
MNWS score	11.1 ± 3.9	11.6 ± 4.7	0.540

Categorical variables are reported as number of patients (%). Continuous variables are shown with (mean ± standard deviation).

FTND: Fagerström Test of Nicotine Dependence, MNWS: Minnesota Nicotine Withdrawal Scale.

Both treatment groups showed no significant difference at the end of the treatment period, but the combination therapy group showed significantly higher abstinence rate during the follow-up period, which took place at 3 and 6 months from the baseline (Table 2). MNWS scores significantly decreased after the treatment in both groups (Table 3), but there was no significant difference between the two groups (p=0.681).

**Table 2:** Comparisons of abstinence rate of the nicotine replacement therapy group and the combination therapy group

	*Nicotine replacement therapy group (n=62 )*	*Combination therapy group (n=86 )*	*p*
Week 6	28 (45.2)	47 (54.7)	0.255
Month 3	20 (32.3)	43 (50.0)	0.031*
Month 6	19 (30.6)	41 (47.7)	0.037*

Categorical variables are reported as number of patients (%). Continuous variables are shown with (mean ± standard deviation).

* p<0.05.

**Table 3:** Changes in Minnesota Nicotine Withdrawal Scale scores before and after the treatment

	*Before*	*After*	*p*
Nicotine replacement therapy group (n=19)	12.8 ± 3.6	6.3 ± 3.4	0.000*
Combination therapy group (n=41)	12.0 ± 4.9	5.9 ± 3.7	0.000*

Categorical variables are reported as number of patients (%). Continuous variables are shown with (mean ± standard deviation).

* p<0.05.

There were no serious adverse events reported during the treatment and follow-up period, and only minor events were reported. In the combination therapy group, 21 subjects from the combination therapy group reported auricle discomfort without swelling, and one participant complained of mild nausea and dizziness that disappeared in a few minutes. A total of 19 subjects reported skin and mouth irritation caused by the nicotine patch and nicotine gum. In all, 84.9% of the combination therapy group stated that the auricular acupuncture treatment was effective in maintaining their abstinence, while the others asserted otherwise.

## DISCUSSION

To our knowledge, the efficacy of the combination therapy of pharmacological intervention and auricular acupuncture for smoking cessation compared to monotherapy has not been studied. NRT, bupropion and varenicline are the first-line pharmacotherapies for smoking cessation^9,10^, as their efficacy has been proven^8^. Using a combination of first-line agents seemed more effective in previous studies, but they also showed more side effects^12,22^. Auricular acupuncture is widely used and well known as a safe alternative intervention for smoking cessation in Korea^23^. In this study, we found that the combination therapy of NRT and auricular acupuncture treatment enhances the abstinence rate significantly compared to NRT alone, without causing any serious adverse events. Also, most of the people in the combination treatment group responded favorably to the auricular acupuncture treatment.

No firm conclusion can be made regarding the effectiveness of acupuncture as a single treatment for smoking cessation according to previous studies. Bier et al.^15^ reported that auricular acupuncture significantly reduces smoking, while Wu et al.^16^ found no significant difference between the auricular acupuncture and sham acupuncture. Fritz et al.^17^ concluded that there is no evidence that the auriculotherapy is superior to placebo. According to a meta-analysis of 25 randomized controlled trials of auricular acupuncture and acupressure, ear acupuncture and acupressure showed superiority over controls^14^. The latest Cochrane reviews in 2014 assert that there is no consistent evidence that acupuncture and acupressure have a sustained benefit on smoking cessation^24^. While the efficacy of auricular acupuncture stands controversial, its safety has been proven as none of the previous studies reported any serious adverse event. Under the circumstances, we researched if auricular acupuncture can play a role as a complementary therapy for smoking cessation.

NRT relieves nicotine withdrawal symptoms by providing nicotine without smoking while the smokers break the habit of smoking^25^. In this study, the nicotine withdrawal symptoms of the smokers were assessed by using the MNWS; however, the MNWS scores of the NRT group and combination therapy group showed no significant difference. This indicates that the acupuncture treatment helped the smokers to quit, through means other than those that alleviate the withdrawal symptoms. The possible mechanisms of the acupuncture treatment are release of endogenous opioid-like substances, such as enkephalin, β-endorphin and endomorphin^26^, and the activation of brain regions related to craving during the abstinence state^27^. Also, the tension relieving effect of auricular acupuncture treatment might have made the counseling more acceptable, and as Bier et al.^15^ reported, auricular acupuncture combined with education and counseling showed a significant effect compared to auricular acupuncture treatment or education alone. More research is required to verify the exact role of the auricular acupuncture treatment for smoking cessation.

Previous studies of auricular acupuncture for smoking cessation showed conflicting results, some claimed that it was no better than sham^16,17^, while others insisted that it was clinically significant^14,15^. Apart from the results, different numbers and choices of acupoints were used in those studies, which indicates that auricular acupuncture treatment for smoking cessation is not standardized. In order for the accumulated data to be credible in the future, the standardization of the auricular acupuncture treatment is essential. We followed the guideline presented by the Association of Korean Medicine^19^, which makes up the core of the smoking cessation programs widely used in Korea^23^. The auricular acupuncture treatment also has an effect for one week, as it stays on the attached site compared to body acupuncture, and this increases the compliance of the smokers. It is not practical for people to visit the clinic three times a week for a twenty minute acupuncture treatment. The auricular acupuncture treatment protocol in this study is safe and easily applicable, and we expect that more research data using the treatment protocol of this study will be available in the future.

### Limitations and strengths

This study took place in a military clinic and all of the subjects were males in their twenties. Therefore, the subjects were short-term smokers with a relatively low nicotine dependence. Also, it was a retrospective study. A prospective, randomized and controlled study with subjects from the general population is needed. The strengths of our study were that it was the first to establish auricular acupuncture as an effective complementary treatment and that the abstinence state was confirmed through urinalysis and not on self-reported abstinence. Also, we have suggested an auricular acupuncture treatment protocol, easy to apply and commonly used in Korea for future research.

## CONCLUSIONS

The results of this study indicate that conventional treatments, such as NRT and behavioral counseling, combined with auricular acupuncture could be a safe and more effective smoking cessation treatment than conventional treatment only.

## CONFLICTS OF INTEREST

Authors have completed and submitted the ICMJE Form for Disclosure of Potential Conflicts of Interest and none was reported.
